# Supplementary material for: Dopamine prediction error signaling in a unique nigrostriatal circuit is critical for associative fear learning
Source: Nat Commun. 2025 Mar 29;16:3066. doi: 10.1038/s41467-025-58382-5 (PMC11954928; doi:10.1038/s41467-025-58382-5)
Supplement: Supplementary file 1 — Supplementary Information [file 41467_2025_58382_MOESM1_ESM.pdf]

Supplementary Figure 1

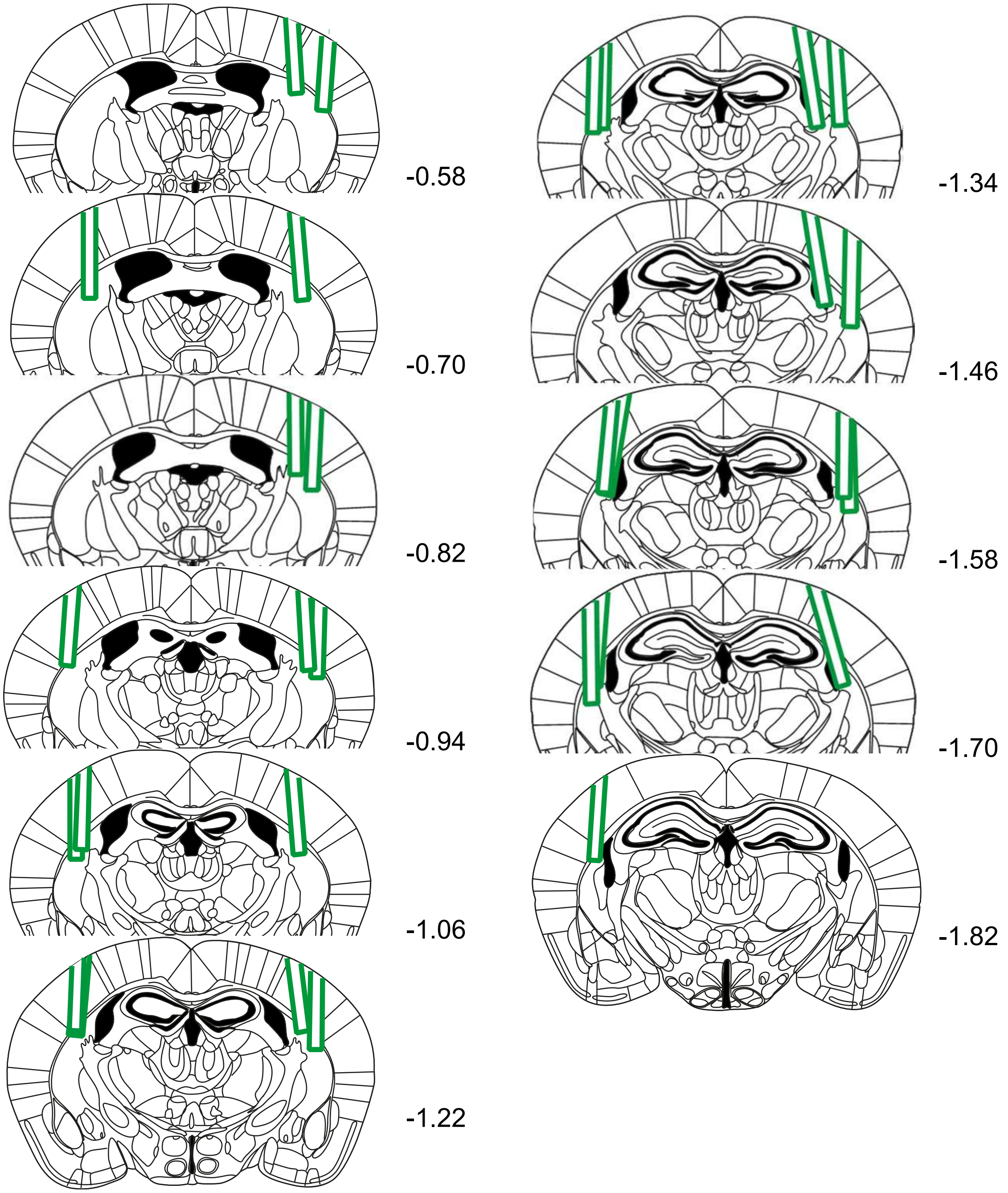

Supplementary Figure 1. Optical fiber placements for GCaMP recordings of DA terminals in TS. Schematic coronal sections showing placement of optical fibers in TS. Numbers represent distance to bregma. Brain schemas are used with permission of Elsevier, from Ref.[96]; permission conveyed through Copyright Clearance Center, Inc.

Supplementary Figure 2

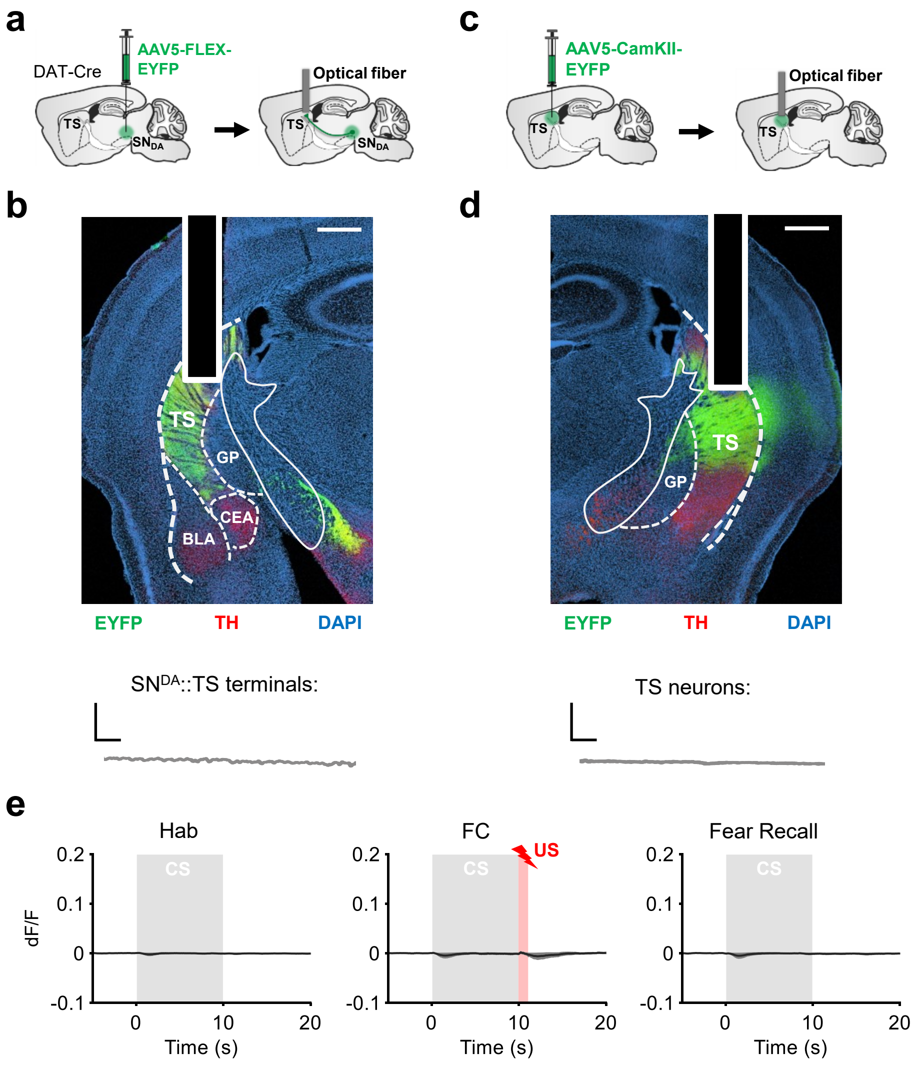

Supplementary Figure 2. Fiber photometry recordings of the control fluorophore. a) Schematic of the surgical procedure showing virus injection in the SN and optical fiber implantation in TS. **b)** Top: example histological image showing expression of control fluorophore EYFP (green) along with immunostaining for tyrosine hydroxylase (TH, red) in the DA terminals and DAPI (blue) staining in TS. White vertical track indicates the optical fiber placement in TS. Scale bar: 0.5 mm. Bottom: example of changes in fluorescence (dF/F) over time in the axon terminals of DA neurons in TS. Scale bar: 10s, 0.05 dF/F. **c)** Schematic of the surgical procedure showing virus injection and optical fiber implantation in TS. **d)** Top: example histological image showing expression of control fluorophore EYFP (green), TH (red) and DAPI (blue) staining in TS. White vertical track indicates the optical fiber placement in TS. Scale bar: 0.5 mm. Bottom: example of changes in fluorescence (dF/F) over time in TS neurons. Scale bar: 10s, 0.01 dF/F. **e)** Average change in fluorescence (n = 4 recording sites) around the time of CS presentation (gray area) during Hab, FC and Fear Recall. The red area during FC represents the US presentation. Shaded regions and error bars represent mean ± s.e.m. Source data are provided as a Source Data file.

Supplementary Figure 3

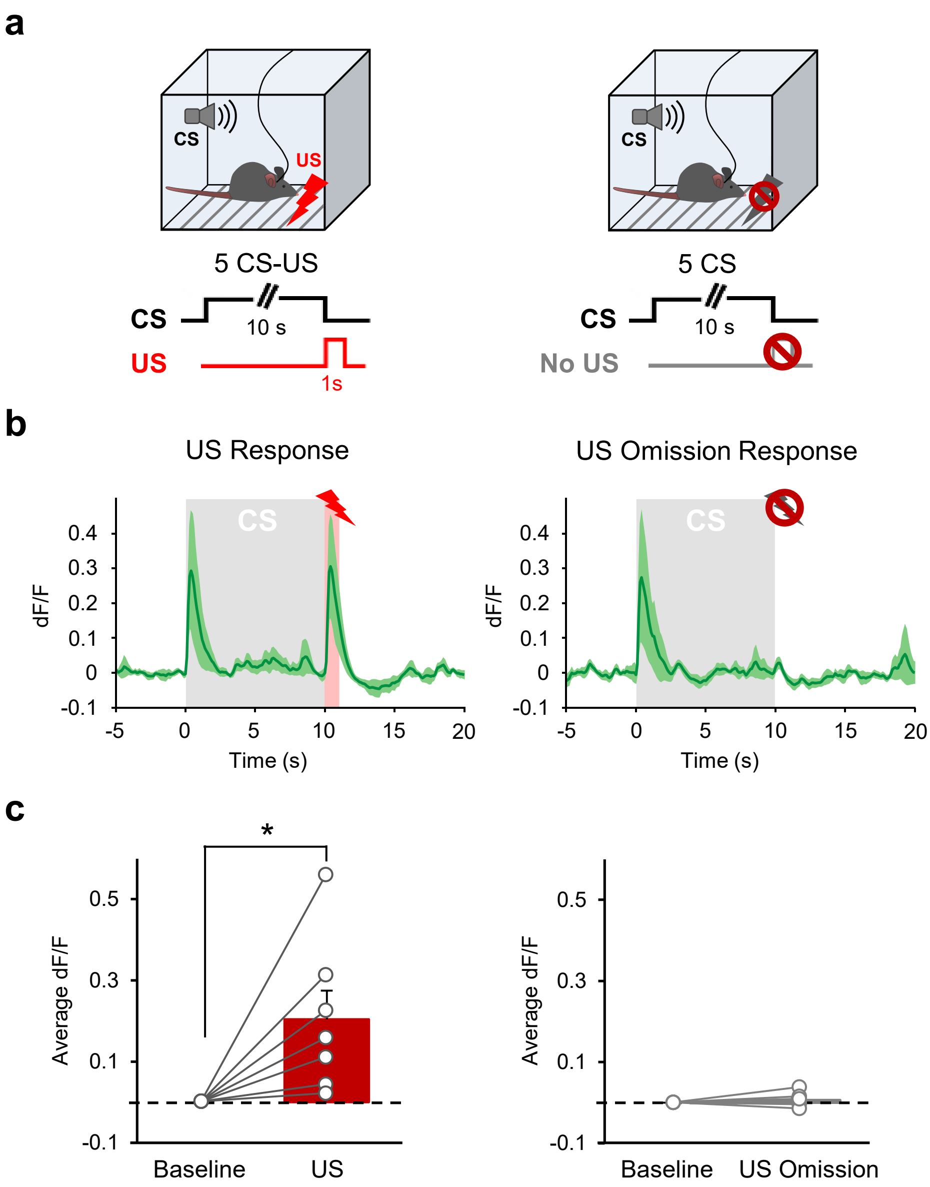

Supplementary Figure 3. DA projections to TS do not respond to omission of the aversive US. a) Schematic of the partial conditioning task where US was omitted randomly in 50% of the trials. Schematic reprinted from ref. 28, copyright (2023), with permission from Elsevier. **b)** Average change in fluorescence (n = 7) around the time of the CS presentation (gray area) during the partial reinforcement session. The red area represents the US presentation. Left: response during trials where US was presented. Right: response during trials where US was omitted. **c)** Left: comparison of average change in fluorescence during the baseline and the US (1s). Note the significant increase in the Ca^{2+} signal during the US presentation (*P = 0.015, two-sided signed-rank test). Right: comparison of average change in fluorescence during the baseline and the US omission (1s). No significant change in the Ca^{2+} signal was observed during US omission (P = 0.46, two-sided signed-rank test). Shaded regions and error bars represent mean ± s.e.m across animals. Source data are provided as a Source Data file.

Supplementary Figure 4

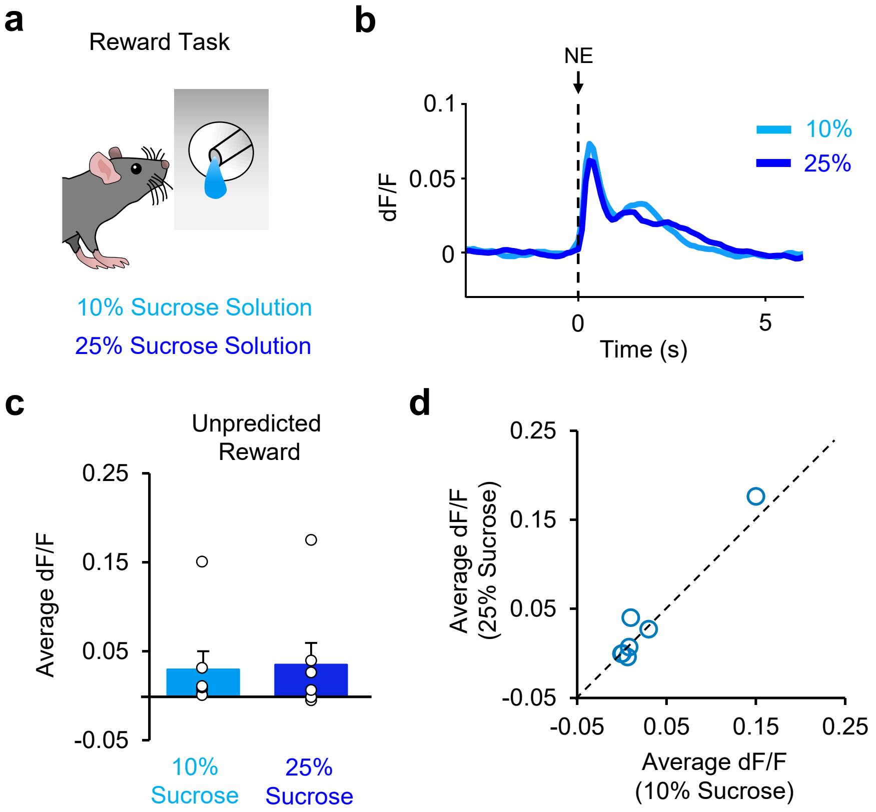

Supplementary Figure 4. Reward responses in DA projections to TS do not scale with the value of reward. a) Schematic of the reward task. Schematic reprinted from ref. 28, copyright (2023), with permission from Elsevier. Animals received reward 50% of the time after entering the noseport. Reward was 10% or 25 % sucrose solution. **b)** Change in fluorescence during rewarded noseport entries (NE) for 10% and 25% sucrose solution in an example animal. **c)** Average change in fluorescence in the 3s after noseport entry during rewarded NE for 10% and 25% sucrose solution. Increasing the value of reward did not affect reward responses (n = 7; P = 0.93, two-sided signed-rank test). **d)** Scatter plot showing responses from each recording site (n = 7) during 10% and 25% sucrose solution presentations. The dashed line indicates the unity line. Note that the data points were close to the unity line indicating that the responses to 10% versus 25% sucrose solution were comparable. Shaded regions and error bars represent mean ± s.e.m across animals. Source data are provided as a Source Data file.

Supplementary Figure 5

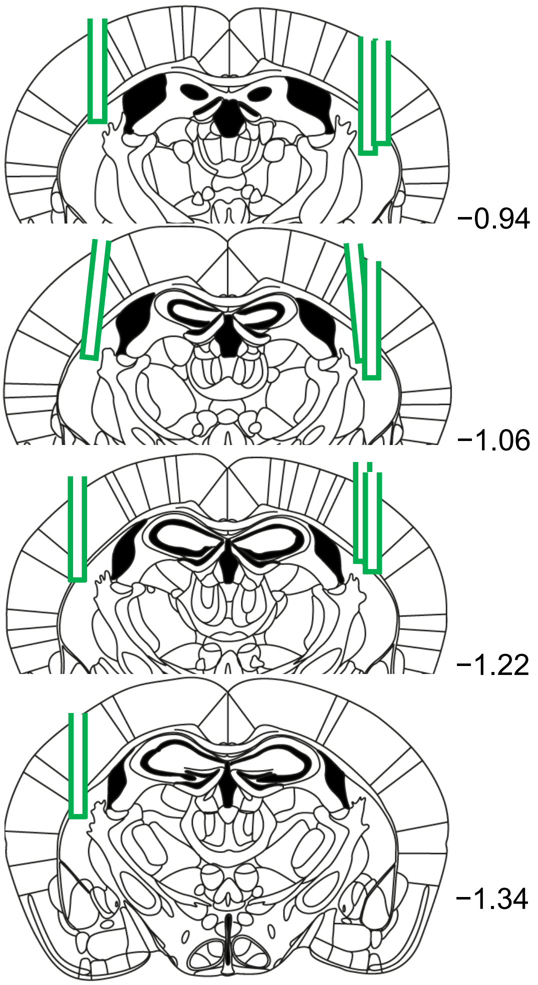

Supplementary Figure 5. Optical fiber placements for dLight recordings in TS. Schematic coronal sections showing placement of optical fibers in TS. Numbers represent distance to bregma. Brain schemas are used with permission of Elsevier, from Ref.[96]; permission conveyed through Copyright Clearance Center, Inc.

Supplementary Figure 6

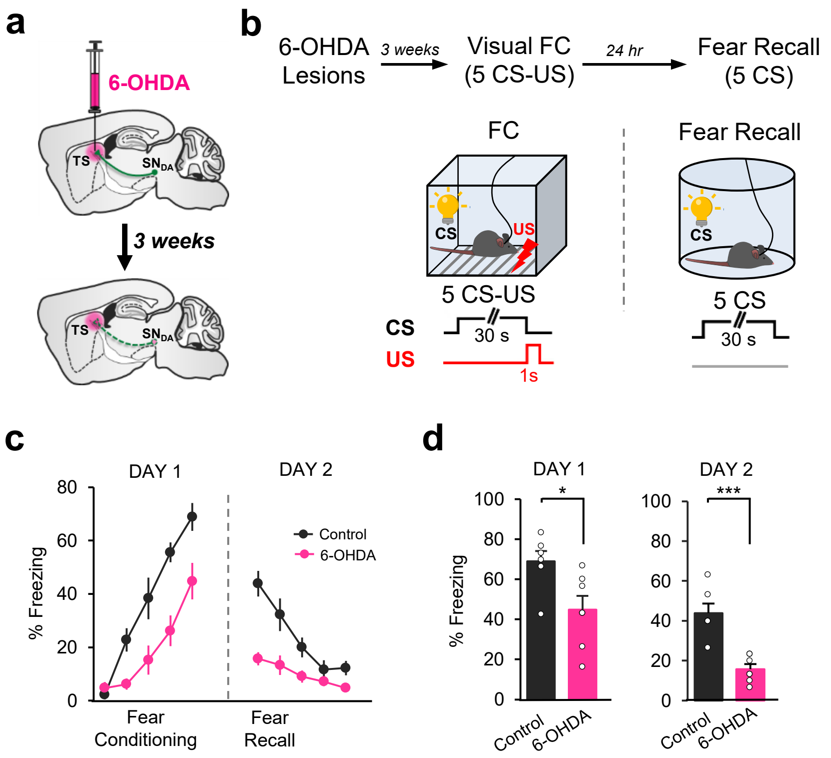

Supplementary Figure 6. TS-projecting DA neurons are required for visual associative fear learning. a) Schematic of the surgical procedure showing 6-OHDA injections in TS (top) and ablation of TS-projecting DA neurons in the SN (SN_{DA}) three weeks later (bottom). **b)** Schematic of the behavioral protocol for 6-OHDA ablation before acquisition of visual fear conditioning. FC: fear conditioning. Schematic reprinted from ref.[28], copyright (2023), with permission from Elsevier. **c)** Behavioral freezing to the CS during FC and fear recall test for mice that received saline (n = 6) and 6-OHDA (n = 7) injections before fear conditioning. 6-OHDA group showed impaired fear acquisition and fear recall (two-way repeated measures ANOVA, fear conditioning: main effect of group: $F_{1,44} = 11.52$, $P = 0.006$; group × trial interaction: $F_{4,44} = 5.09$, $P = 0.0018$; fear recall: main effect of group: $F_{1,44} = 13.10$, $P = 0.004$; group × trial interaction: $F_{4,44} = 7.01$, $P = 0.0002$). **d)** Left: freezing to the last CS during fear conditioning. 6-OHDA group froze significantly less compared to saline-injected control group (two-sided t-test, $t(11) = 2.66$, $P = 0.02$). Right: freezing to the CS at the beginning of fear recall (first CS). 6-OHDA group froze significantly less compared to control group (two-sided t-test, $t(11) = 5.13$, $P = 0.0003$). Error bars represent mean ± s.e.m. across animals. Source data are provided as a Source Data file.

Supplementary Figure 7

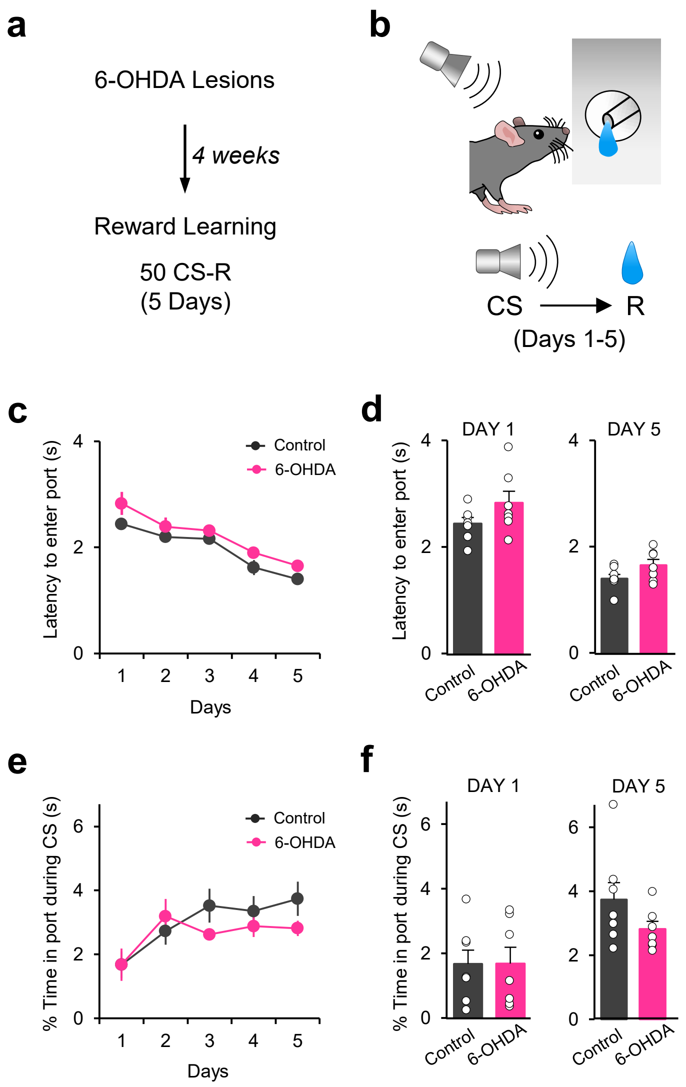

Supplementary Figure 7. Ablation of TS-projecting DA neurons does not impair associative reward learning. a) Schematic of the behavioral protocol for 6-OHDA ablation before reward learning. **b)** Schematic of the reward learning task. Schematic reprinted from ref.[28], copyright (2023), with permission from Elsevier. **c)** Latency to enter the port during CS over the course of the reward task for control (n = 7) and 6-OHDA (n =7) groupd. **d)** Latency to enter the port during the first and last days of the reward task. The two groups (control, n = 7; 6-OHDA, n = 7) exhibited comparable performance (Day 1: two-sided t-test, $t(12) = 1.56$, $P = 0.14$; Day 5: two-sided t-test, $t(12) = 1.84$, $P = 0.09$). **e)** Percent time animals spent in the port during CS over the course of the reward task. **f)** Percent time animals spent in the port during the first and last days of the reward task. The two groups (control, n = 7; 6-OHDA, n = 7) exhibited comparable performance (Day 1: two-sided t-test, $t(12) = 0.01$, $P = 0.99$; Day 5: two-sided t-test, $t(12) = 1.49$, $P = 0.16$). Error bars represent mean ± s.e.m. across animals. Source data are provided as a Source Data file.

Supplementary Figure 8

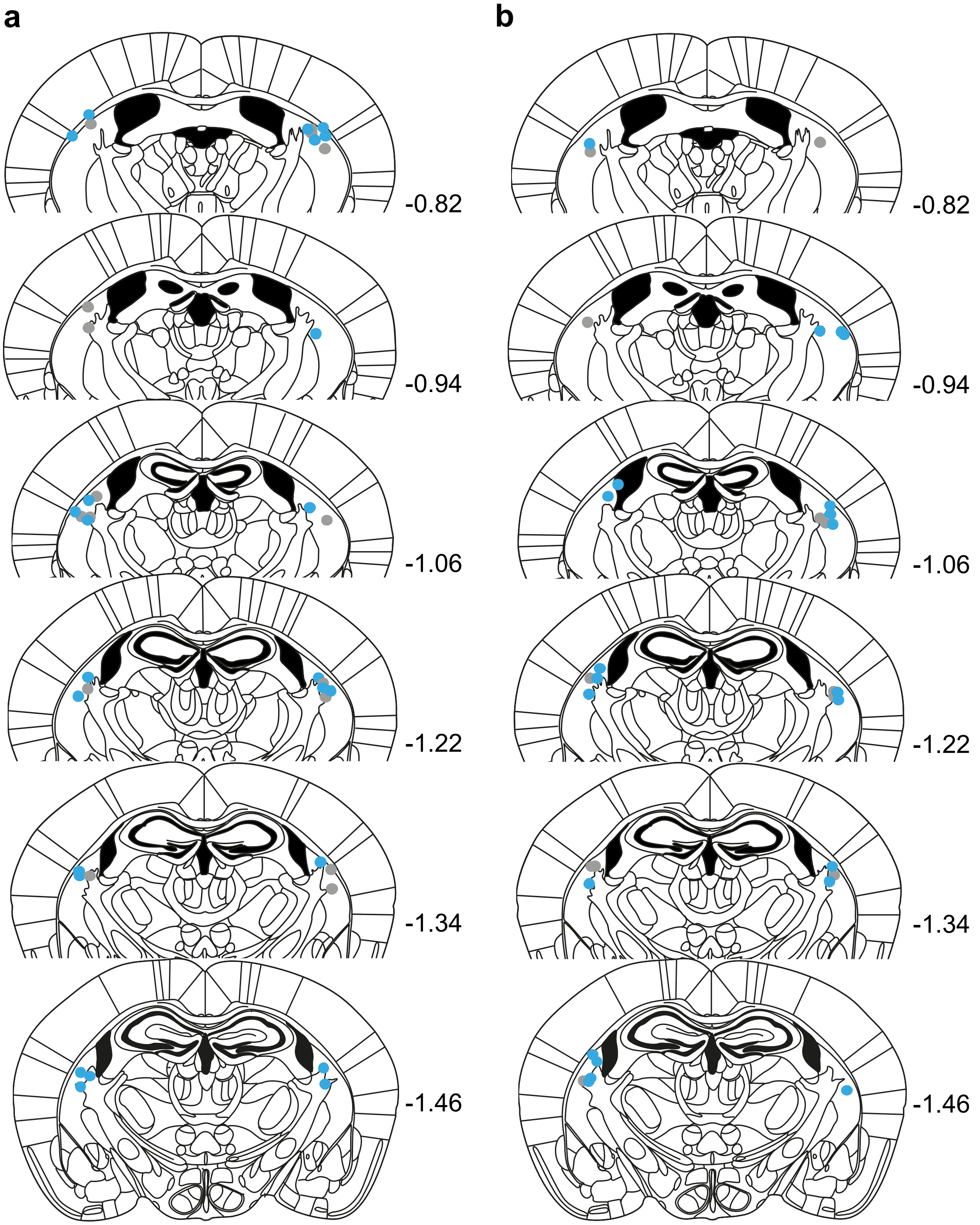

Supplementary Figure 8. Optical fiber placements for optogenetic excitation of DA terminals in TS. Schematic coronal sections showing placement of optical fiber tips in TS for ChR2 (blue) and EYFP (gray) mice in US-Paired and No-US groups **(a)** and ChR2 (blue) and EYFP (gray) mice in CS-Paired and ITI groups **(b)**. Numbers represent distance (mm) to bregma. Brain schemas are used with permission of Elsevier, from Ref.[96]; permission conveyed through Copyright Clearance Center, Inc.

Supplementary Figure 9

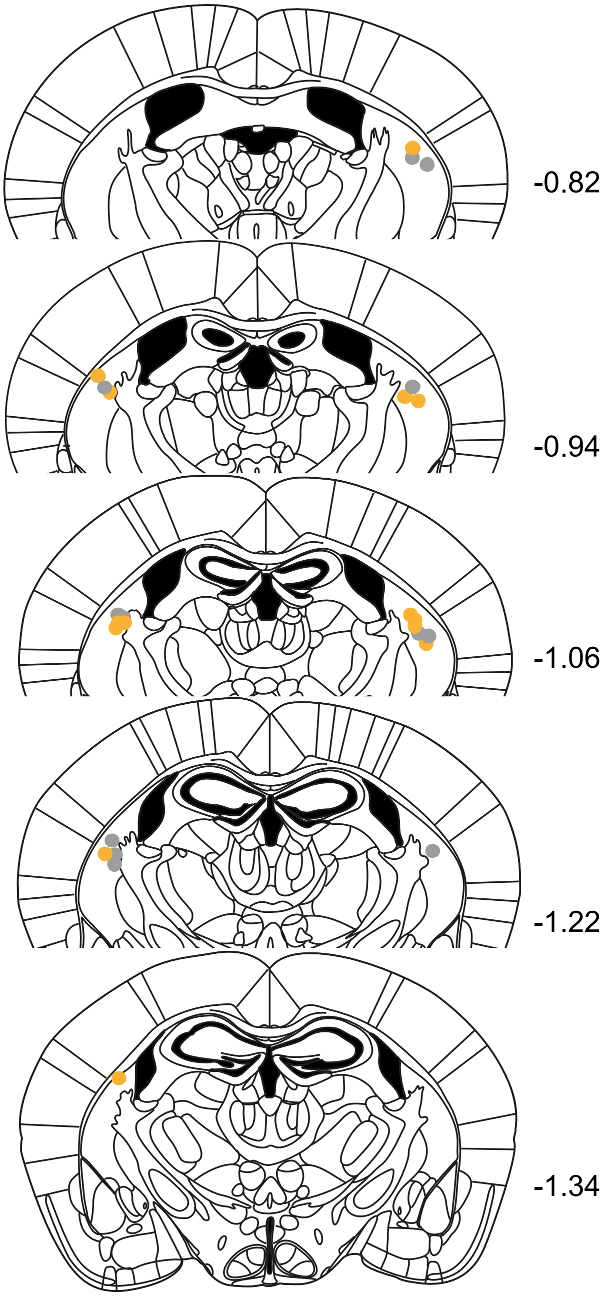

Supplementary Figure 9. Optical fiber placements for optogenetic inhibition of DA terminals in TS. Schematic coronal sections showing placement of optical fiber tips in TS for eArch (yellow) and EYFP control (gray) groups. Numbers represent distance to bregma. Brain schemas are used with permission of Elsevier, from Ref.[96]; permission conveyed through Copyright Clearance Center, Inc.

Supplementary Figure 10

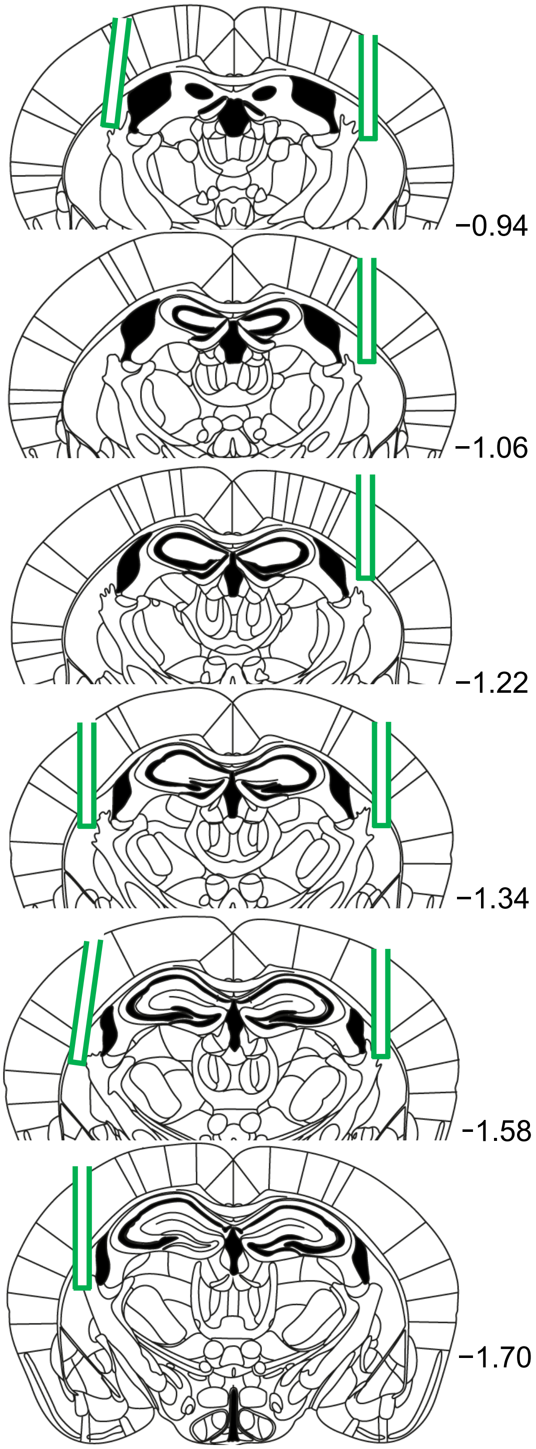

Supplementary Figure 10. Optical fiber placements for GCaMP recordings in TS. Schematic coronal sections showing placement of optical fibers in TS. Numbers represent distance to the bregma. Brain schemas are used with permission of Elsevier, from Ref.[96]; permission conveyed through Copyright Clearance Center, Inc.

Supplementary Figure 11

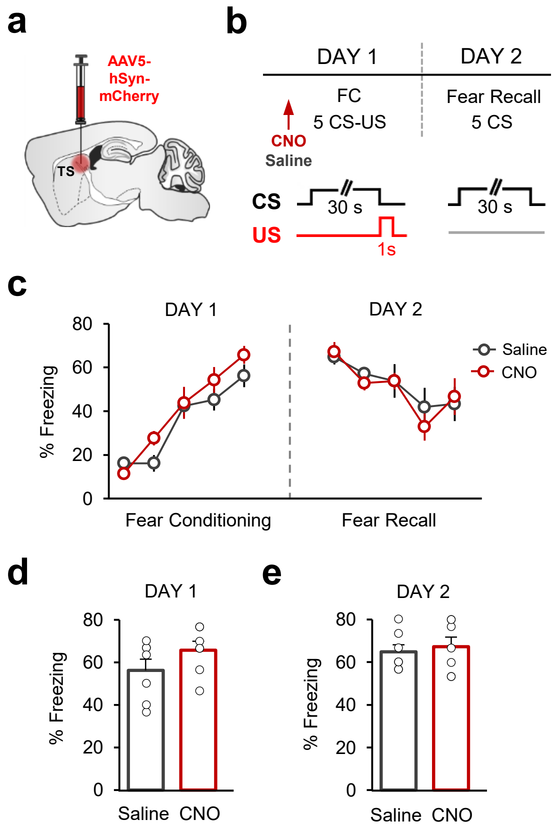

Supplementary Figure 11. Effect of CNO on fear learning depends on expression of DREADDs in TS. a) Schematic of the surgical procedure showing control virus injection in TS. **b)** Top: schematic of the behavioral protocol. FC: fear conditioning. Bottom: schematic of CS and US presentations during FC and Fear Recall session. **c)** Percent freezing to the CS during FC and fear recall session for saline- (gray, n = 7) and CNO-injected mice (red, n = 7). The two groups exhibited comparable freezing levels during FC (two-way repeated measures ANOVA, no main effect of group, $F_{1,48} = 1.21$, $P = 0.29$ and group × trial interaction, $F_{4,48} = 1.8$, $P = 0.14$) and fear recall (two-way repeated measures ANOVA, no main effect of group, $F_{1,48} = 0.04$, $P = 0.82$ and group × trial interaction, $F_{4,48} = 0.73$, $P = 0.57$), indicating that CNO injection did not have an effect on fear learning in control mice expressing mCherry in TS. **d)** Freezing to the last CS during fear conditioning. **e)** Freezing to the first CS during fear recall. Error bars represent mean ± s.e.m. across animals. Source data are provided as a Source Data file.

Supplementary Figure 12

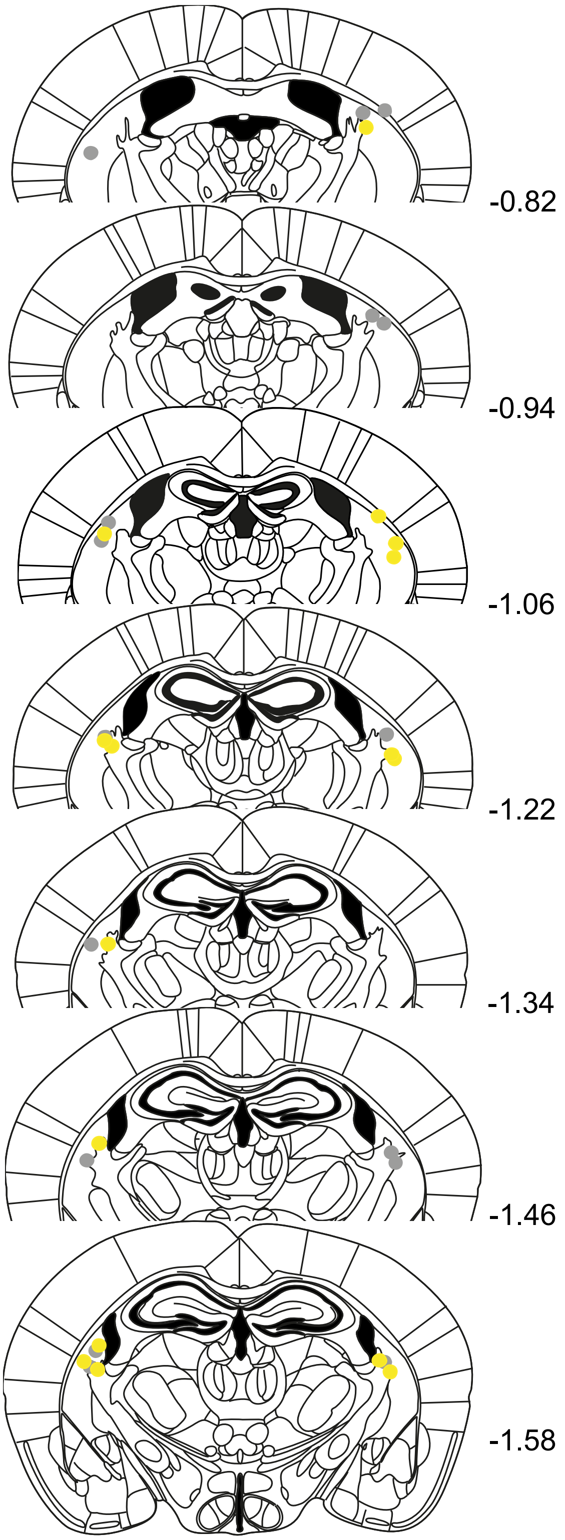

Supplementary Figure 12. Optical fiber placements for optogenetic inhibition of TS neurons. Schematic coronal sections showing placement of optical fiber tips in TS for eArch (yellow) and EYFP control (gray) group. Numbers represent distance to bregma. Brain schemas are used with permission of Elsevier, from Ref.[96]; permission conveyed through Copyright Clearance Center, Inc.

Supplementary Figure 13

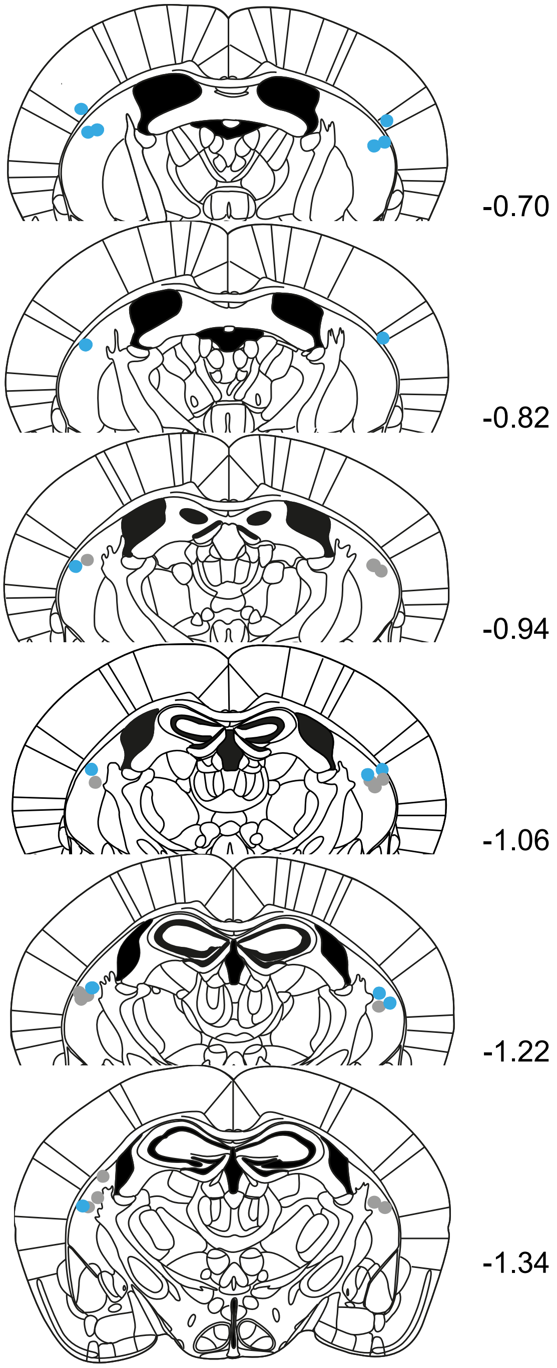

Supplementary Figure 13. Optical fiber placements for optogenetic excitation of TS neurons. Schematic coronal sections showing placement of optical fiber tips in TS for ChR2 (blue) and EYFP control (gray) group. Numbers represent distance to bregma. Brain schemas are used with permission of Elsevier, from Ref.[96]; permission conveyed through Copyright Clearance Center, Inc.

Supplementary Figure 14

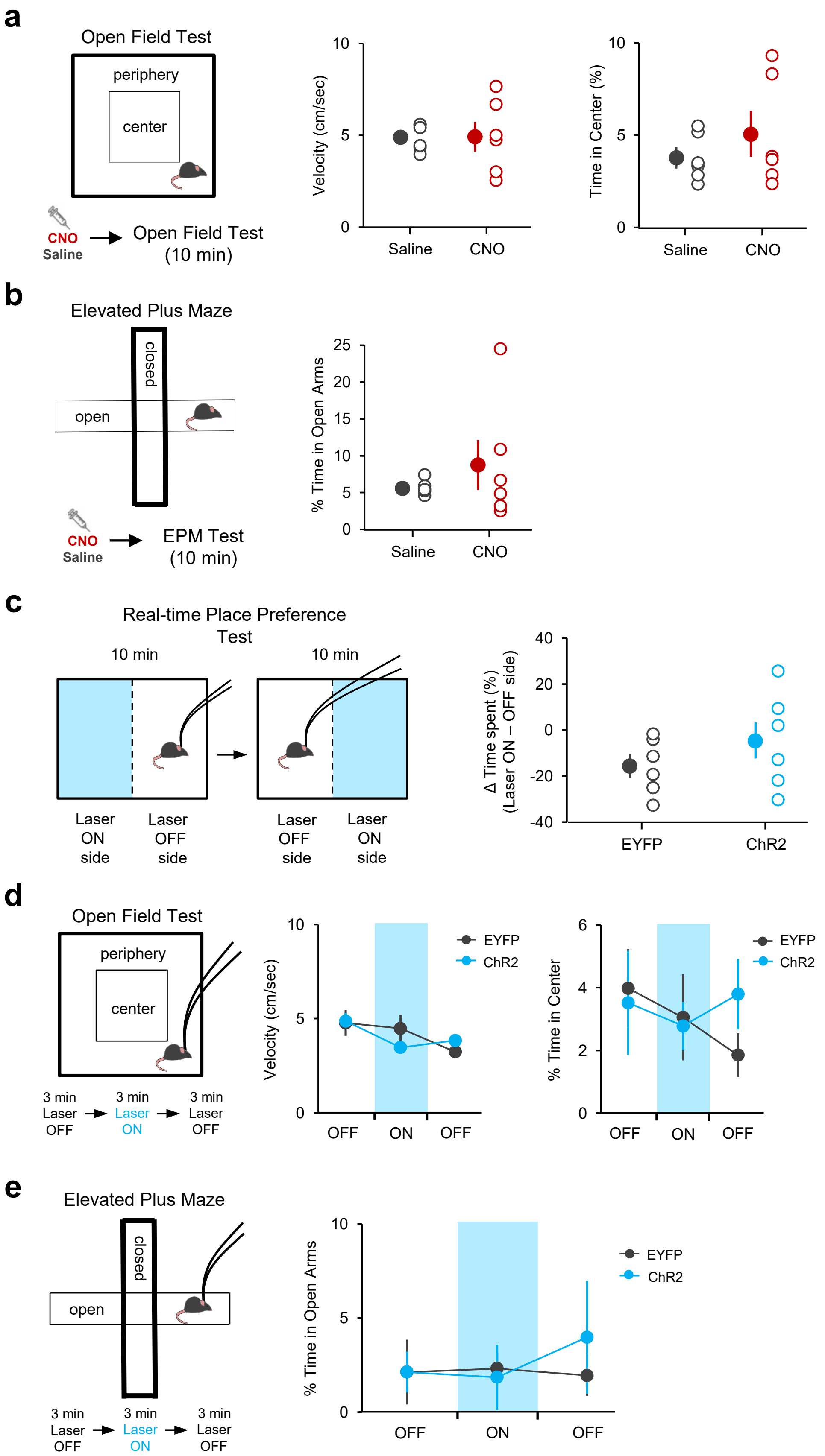

Supplementary Figure 14. Manipulation of neuronal activity in TS does not effect movement, anxiety-like behaviors and real-time place preference. a) Left: schematic of the open field test. Velocity (middle) and percent time mice spent in the center of the open field (right) for saline (n = 6) and CNO (n = 6) groups. The two groups behaved comparably (two-sided t-tests, t(10) = 0.03, P = 0.96; t(10) = 0.96, P = 0.35). **b)** Left: schematic of the elevated plus maze test. Right: percent time mice spent in the open arms of the elevated plus maze for saline (n = 6) and CNO (n = 6) groups. The two groups behaved comparably (two-sided t-test, t(10) = 0.93, P = 0.37). **c)** Left: schematic of the real-time place preference test. Right: difference between the percent of time mice (EYFP, n = 6; ChR2, n = 6) spent in laser ON minus laser OFF side (two-sided t-test, t(10) = 1.11, P = 0.29). **d)** Left: schematic of the open field test. Middle: velocity of mice (EYFP, n = 6; ChR2, n = 6) in the open field did not differ between the groups (two-way repeated measures ANOVA, no main effect of group: $F_{1,20}$ = 0.05, P = 0.81). Right: Percent time mice spent in the center of the open field did not differ between the groups (two-way repeated measures ANOVA, no main effect of group: $F_{1,20}$ = 0.13, P = 0.71). **e)** Percent time mice (EYFP, n = 6; ChR2, n = 6) spent in the open arms of the elevated plus maze during laser ON and OFF epochs. The two groups behaved comparably (two-way repeated measures ANOVA, no main effect of group: $F_{1,20}$ = 0.11, P = 0.74). Error bars represent mean ± s.e.m. across animals. Source data are provided as a Source Data file.

Supplementary Figure 15

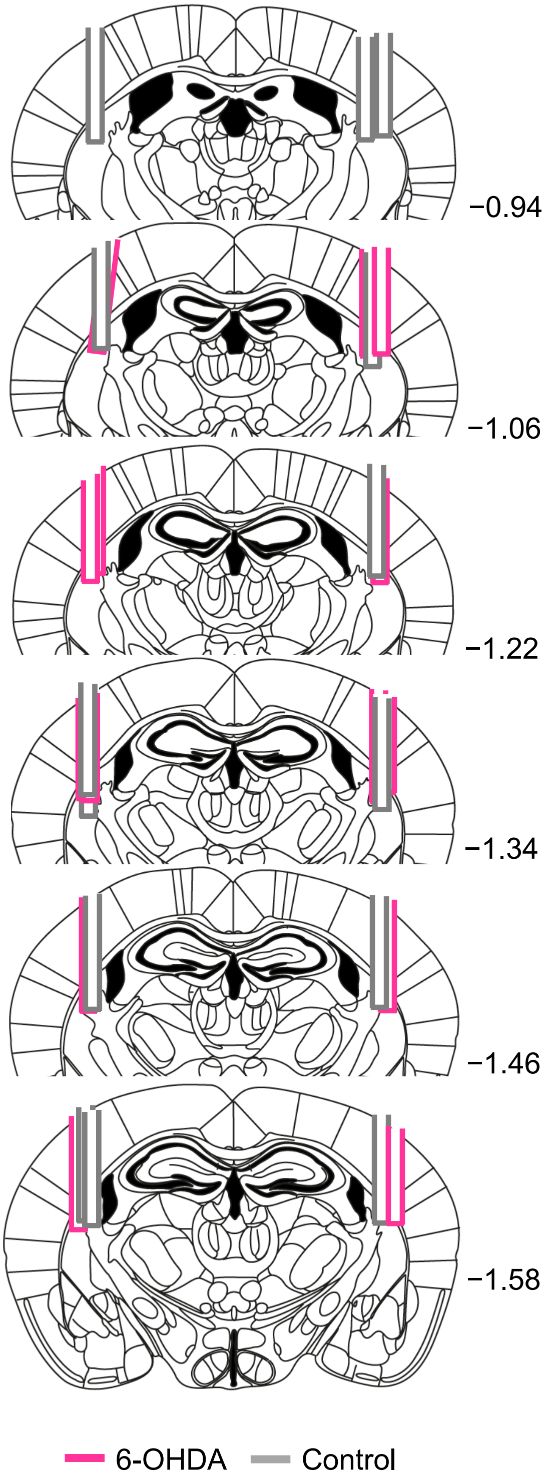

Supplementary Figure 15. Optical fiber placements for GCaMP recordings in the TS following 6-OHDA Lesions of TS-projecting DA neurons. Schematic coronal sections showing placement of optical fibers in the TS for 6-OHDA (pink) and Control (gray) groups. Numbers represent distance to bregma. Brain schemas are used with permission of Elsevier, from Ref.[96]; permission conveyed through Copyright Clearance Center, Inc.
