# Supplementary material for: Dopamine prediction error signaling in a unique nigrostriatal circuit is critical for associative fear learning
Source: Nat Commun. 2025 Mar 29;16:3066. doi: 10.1038/s41467-025-58382-5 (PMC11954928; doi:10.1038/s41467-025-58382-5)
Supplement: Supplementary file 2 — Reporting Summary [file 41467_2025_58382_MOESM2_ESM.pdf]

nature portfolio

Corresponding author(s): Sevil Duvarci

Last updated by author(s): 27/01/25

Reporting Summary

Nature Portfolio wishes to improve the reproducibility of the work that we publish. This form provides structure for consistency and transparency in reporting. For further information on Nature Portfolio policies, see our Editorial Policies and the Editorial Policy Checklist.

Statistics

For all statistical analyses, confirm that the following items are present in the figure legend, table legend, main text, or Methods section.

n/a	Confirmed	
☐	☒	The exact sample size (*n*) for each experimental group/condition, given as a discrete number and unit of measurement
☐	☒	A statement on whether measurements were taken from distinct samples or whether the same sample was measured repeatedly
☐	☒	The statistical test(s) used AND whether they are one- or two-sided<br>*Only common tests should be described solely by name; describe more complex techniques in the Methods section.*
☒	☐	A description of all covariates tested
☐	☒	A description of any assumptions or corrections, such as tests of normality and adjustment for multiple comparisons
☐	☒	A full description of the statistical parameters including central tendency (e.g. means) or other basic estimates (e.g. regression coefficient) AND variation (e.g. standard deviation) or associated estimates of uncertainty (e.g. confidence intervals)
☐	☒	For null hypothesis testing, the test statistic (e.g. *F*, *t*, *r*) with confidence intervals, effect sizes, degrees of freedom and *P* value noted<br>*Give P values as exact values whenever suitable.*
☒	☐	For Bayesian analysis, information on the choice of priors and Markov chain Monte Carlo settings
☒	☐	For hierarchical and complex designs, identification of the appropriate level for tests and full reporting of outcomes
☒	☐	Estimates of effect sizes (e.g. Cohen's *d*, Pearson's *r*), indicating how they were calculated

Our web collection on statistics for biologists contains articles on many of the points above.

Software and code

Policy information about availability of computer code

Data collection	Fiber photometry data were acquired using Cheetah acquisition software, Neuralynx.
Data analysis	Data analysis was performed using Matlab (Mathworks). No original code was used in the analysis of the data. Statistical analysis was performed using GraphPad Prism (GraphPad Software) and Matlab (Mathworks).

For manuscripts utilizing custom algorithms or software that are central to the research but not yet described in published literature, software must be made available to editors and reviewers. We strongly encourage code deposition in a community repository (e.g. GitHub). See the Nature Portfolio guidelines for submitting code & software for further information.

Data

Policy information about availability of data

All manuscripts must include a data availability statement. This statement should provide the following information, where applicable:

- Accession codes, unique identifiers, or web links for publicly available datasets
- A description of any restrictions on data availability
- For clinical datasets or third party data, please ensure that the statement adheres to our policy

Source data is provided with this paper. Raw data will be available from the corresponding author upon reasonable request.

Research involving human participants, their data, or biological material

Policy information about studies with human participants or human data. See also policy information about sex, gender (identity/presentation), and sexual orientation and race, ethnicity and racism.

Reporting on sex and gender	N/A
Reporting on race, ethnicity, or other socially relevant groupings	N/A
Population characteristics	N/A
Recruitment	N/A
Ethics oversight	N/A

Note that full information on the approval of the study protocol must also be provided in the manuscript.

Field-specific reporting

Please select the one below that is the best fit for your research. If you are not sure, read the appropriate sections before making your selection.

☒ Life sciences ☐ Behavioural & social sciences ☐ Ecological, evolutionary & environmental sciences

For a reference copy of the document with all sections, see nature.com/documents/nr-reporting-summary-flat.pdf

Life sciences study design

All studies must disclose on these points even when the disclosure is negative.

Sample size	No statistical methods were used to predetermine sample size, but our sample sizes are similar to those generally used in the fear conditioning field. Sample sizes were chosen to ensure robustness of findings while keeping the number of experimental animals to the necessary minimum, in accordance with 3R principles.
Data exclusions	Animals with incorrect optical fiber placements and that failed to show adequate virus expression were excluded from analysis.
Replication	All results were obtained using groups of mice that were ran in several cohorts. All cohorts exhibited comparable results and all analysis was performed on the combined data across cohorts.
Randomization	Animals were randomly assigned to experimental groups after ensuring that all experimental groups were matched for age. For 6-OHDA lesioning, optogenetic and chemogenetic experiments, littermate mice were assigned to experimental versus control groups.
Blinding	For 6-OHDA lesion experiments, investigators were blinded to group allocation during data collection. For other experiments, investigators were not blinded during data collection. Behavioral analysis (scoring of behavioral freezing) was performed blind to the experimental condition.

Reporting for specific materials, systems and methods

We require information from authors about some types of materials, experimental systems and methods used in many studies. Here, indicate whether each material, system or method listed is relevant to your study. If you are not sure if a list item applies to your research, read the appropriate section before selecting a response.

Materials & experimental systems

n/a	Involved in the study	
☐	☒	Antibodies
☐	☐	Eukaryotic cell lines
☐	☐	Palaeontology and archaeology
☐	☒	Animals and other organisms
☐	☐	Clinical data
☐	☐	Dual use research of concern
☐	☐	Plants

Methods

n/a	Involved in the study	
☐	☐	ChIP-seq
☐	☐	Flow cytometry
☐	☐	MRI-based neuroimaging

Antibodies

Antibodies used	Primary antibodies: polyclonal rabbit anti-tyrosine hydroxylase (Catalog # 657012, Thermo Fisher), monoclonal mouse anti-TH (catalog # MAB318, Millipore), polyclonal guinea pig anti-TH (catalog # 213004, Synaptic Systems), polyclonal rabbit anti-GFP (catalog # A11122, Life Technology), polyclonal chicken anti-GFP (catalog # AB13970, Abcam), and mCherry monoclonal anti-rat (catalog # M11217, Thermo Fisher).<br>Secondary antibodies: Alexa Fluor 568 goat anti-rabbit (catalog # A11011, Thermo Fisher), Alexa Fluor 568 goat anti-mouse (catalog # A11004, Thermo Fisher), Alexa Fluor 568 goat anti-guinea pig (catalog # A11075, Thermo Fisher), Alexa Fluor 405 goat anti-rabbit (catalog # A31556, Thermo Fisher), Alexa Fluor 488 goat anti-rabbit (catalog # A11008, Thermo Fisher), Alexa Fluor 488 goat anti-chicken (catalog # AB150173, Abcam), Alexa Fluor 568 goat anti-rat (catalog # A11077, Thermo Fisher).
Validation	All antibodies used in this study were validated for use in mice by the companies that provided them and this information is available on their websites.

Eukaryotic cell lines

Policy information about cell lines and Sex and Gender in Research

Cell line source(s)	N/A
Authentication	N/A
Mycoplasma contamination	N/A
Commonly misidentified lines (See ICLAC register)	N/A

Palaeontology and Archaeology

Specimen provenance	N/A
Specimen deposition	N/A
Dating methods	N/A

☐ Tick this box to confirm that the raw and calibrated dates are available in the paper or in Supplementary Information.

Ethics oversight	N/A

Note that full information on the approval of the study protocol must also be provided in the manuscript.

Animals and other research organisms

Policy information about studies involving animals; ARRIVE guidelines recommended for reporting animal research, and Sex and Gender in Research

Laboratory animals	C57Bl/6N mice and DAT-Cre mice (backcrossed with C57Bl/6N) were used in this study.
Wild animals	No wild animals were used.
Reporting on sex	Due to the time-intensive nature of the experiments and limited resources, only male mice were used in the study and a comparison between sexes was not performed.
Field-collected samples	N/A
Ethics oversight	All procedures were conducted in accordance with the guidelines of the German Animal Protection Act and were approved by the local authorities (Regierungspräsidium Darmstadt).

Note that full information on the approval of the study protocol must also be provided in the manuscript.

Clinical data

Policy information about clinical studies

All manuscripts should comply with the ICMJE guidelines for publication of clinical research and a completed CONSORT checklist must be included with all submissions.

Clinical trial registration	N/A

Study protocol	N/A
Data collection	N/A
Outcomes	N/A

Dual use research of concern

Policy information about [dual use research of concern]

Hazards

Could the accidental, deliberate or reckless misuse of agents or technologies generated in the work, or the application of information presented in the manuscript, pose a threat to:

No	Yes	
☒	☐	Public health
☒	☐	National security
☒	☐	Crops and/or livestock
☒	☐	Ecosystems
☒	☐	Any other significant area

Experiments of concern

Does the work involve any of these experiments of concern:

No	Yes	
☒	☐	Demonstrate how to render a vaccine ineffective
☒	☐	Confer resistance to therapeutically useful antibiotics or antiviral agents
☒	☐	Enhance the virulence of a pathogen or render a nonpathogen virulent
☒	☐	Increase transmissibility of a pathogen
☒	☐	Alter the host range of a pathogen
☒	☐	Enable evasion of diagnostic/detection modalities
☒	☐	Enable the weaponization of a biological agent or toxin
☒	☐	Any other potentially harmful combination of experiments and agents

Plants

Seed stocks	N/A
Novel plant genotypes	N/A
Authentication	N/A

ChIP-seq

Data deposition

☐ Confirm that both raw and final processed data have been deposited in a public database such as GEO.

☐ Confirm that you have deposited or provided access to graph files (e.g. BED files) for the called peaks.

Data access links<br>*May remain private before publication.*	N/A
Files in database submission	N/A

Genome browser session
(e.g. UCSC)

N/A

Methodology

Replicates	N/A
Sequencing depth	N/A
Antibodies	N/A
Peak calling parameters	N/A
Data quality	N/A
Software	N/A

Flow Cytometry

Plots

Confirm that:

- ☐ The axis labels state the marker and fluorochrome used (e.g. CD4-FITC).
- ☐ The axis scales are clearly visible. Include numbers along axes only for bottom left plot of group (a 'group' is an analysis of identical markers).
- ☐ All plots are contour plots with outliers or pseudocolor plots.
- ☐ A numerical value for number of cells or percentage (with statistics) is provided.

Methodology

Sample preparation	N/A
Instrument	N/A
Software	N/A
Cell population abundance	N/A
Gating strategy	N/A

☐ Tick this box to confirm that a figure exemplifying the gating strategy is provided in the Supplementary Information.

Magnetic resonance imaging

Experimental design

Design type	N/A
Design specifications	N/A
Behavioral performance measures	N/A

Acquisition

Imaging type(s)	N/A
Field strength	N/A
Sequence & imaging parameters	N/A
Area of acquisition	N/A

Diffusion MRI ☐ Used ☐ Not used

Preprocessing

Preprocessing software	N/A
Normalization	N/A
Normalization template	N/A
Noise and artifact removal	N/A
Volume censoring	N/A

Statistical modeling & inference

Model type and settings	N/A
Effect(s) tested	N/A

Specify type of analysis: ☐ Whole brain ☐ ROI-based ☐ Both

Statistic type for inference (See Eklund et al. 2016)	N/A
Correction	N/A

Models & analysis

n/a	Involved in the study
☐	☐ Functional and/or effective connectivity
☐	☐ Graph analysis
☐	☐ Multivariate modeling or predictive analysis

Functional and/or effective connectivity	N/A
Graph analysis	N/A
Multivariate modeling and predictive analysis	N/A
